# Work–Life Balance: How Can We Achieve It within the Work Environment?

**DOI:** 10.3389/fped.2016.00040

**Published:** 2016-05-02

**Authors:** Harissios Vliagoftis

**Affiliations:** ^1^Pulmonary Research Group, Department of Medicine, University of Alberta, Edmonton, AB, Canada

**Keywords:** work–life balance, academic medicine, work demands, personal approach, training

During more than 30 years since I enrolled into Medical School, I have had discussions around work–life balance with many friends, colleagues, and trainees. It appears to me that as I am getting older, I have these discussions more frequently, and also that more and more of my colleagues and trainees complain about a lack of work–life balance and are unhappy with the demands of the job on their daily lives. I am not sure if this subjective observation reflects changes in my work environment or if we as a society are changing attitudes toward work and life priorities, but we cannot find ways to alter our work demands to reflect these changes.

As is customary in “philosophical” texts, I was looking for quotes from famous people, which would support my personal approach to work–life balance. Since I am Greek, I was looking for quotes from ancient Greek philosophers, quotes we encounter often in our readings although very few of us, I suspect, have ever read the original documents. I could not find any quote that I could be certain that was reflecting the opinions expressed in the original document and then remembered a quote from a contemporary book I read, “This is antiquity’s value; it could be used to sanction anything. … bring in a few words from the writings (those denoised sound bites) of a Greek whose bones are well-mineralized and you have put wheels on your stupidity” (1). To avoid sounding stupid, or misrepresenting the writings of ancient Greek philosophers, I will continue without any such sound bites, but I will discuss here my experiences from my formative years in Greece that I believe have played a large role in my decision not only to follow a career in academic medicine but also to develop my personal approach to work–life balance.

As a medical student at the Aristotelian University of Thessaloniki, Greece, I had the good fortune to do research with members of a small non-university-based, but with a very academic philosophy, research group within a specialty hospital in Greece. My interactions with the members of that group and my integration into the group had a significant influence in my personal and scientific growth. Many, if not most, of my mentors from that time are not active scientifically anymore, but I would be very happy if they saw this narrative and felt proud to have helped a young student find his way in science and in life.

The group was doing high-quality research with little resources and little external support. All members of the group were in that environment because they loved doing research and wanted to feed their curiosity through their studies. People worked very well together in an open and friendly work environment. What was very interesting was that the members of the group were not only friendly to each other but were friends and this attitude extended not only to people working within this small group but also to other people working within the same hospital. They were all people with families and friends outside the work place, but they also found the work environment enjoyable and rewarding and interactions with their colleagues as valuable as interactions with any other friends. My mentors and co-workers back then found the work environment so enjoyable that that they would willingly extend their day or come back to “live” in the work environment in the evenings. The people gathering almost daily included the director of the group, a number of senior members, medical and non-medical personnel affiliated with the hospital housing the group, and a number of trainees at all levels of training. During those evening gatherings at the work place, discussions ranged from science to culture to personal problems to issues that may not be appropriate to be discussed here.

In that environment, I learned to appreciate what science could offer and learned the importance of general knowledge and involvement with issues ranging from science to politics to the daily problems of the community to sports. Learned that you can discuss these issues in an open environment with people who want to hear and value your opinion and support you no matter how different is your opinion from that of the senior or influential members of the group. I also learned that you do not have to separate completely your life from your work, the work environment can offer much more than work satisfaction, can offer friends, and a place to have fun and even discuss your personal problems. I learned that working with “friends” and enjoying what you do helps you develop work–life balance by having work be large part of your life without consuming all your waking time. I believed back then, and still believe now, that those people had found an excellent way to attain work–life balance, and as a medical student, I thought that I would be very lucky if I could find myself working in such a place later in my life.

My tenure in that environment led to opportunities to become more involved with research during my subsequent training and had a major role in my decision to follow an academic career. Today I am a clinician scientist, a specialist in clinical immunology and allergy with a heavy research load. I am a researcher, I am a clinician, I am an educator with responsibilities for my graduate and postgraduate students, and lately, I am also an administrator with responsibilities for my division. My daily involvement with all these activities, activities that often require different skill sets and different approaches to problem solving, makes it sometimes difficult for me to know whether on a given day I was able to do anything productive at all and whether I was able to fulfill my role as a university professor. On those days, I try to re-evaluate my approaches and see how I can attain the work–life balance I saw in my mentors long time ago.

Poor work–life balance can have both physical and mental health consequences. Men and women reporting a poor work–life balance also report poor well-being, more sickness absences, and more health problems (2, 3) compared to people who experience better work–life balance. There has been a lot of discussion and many publications addressing the difficulty in achieving work–life balance in academic medicine. It appears to me that some of the barriers we face to achieve work–life balance may be different from that of other professions, and according to academic physicians themselves, some of these barriers are deeply rooted within our professional culture (4). First of all, we need to accommodate the demands of three jobs to fulfill our clinical, teaching, and research missions. In addition, our profession was for too long dominated by colleagues who considered anything less than complete focus on work demands inappropriate for a physician. It is encouraging that these attitudes are changing, and taking advantage of programs that may allow better work–life balance, such as flexible hours, working from home, or easier access to time off for personal issues, is not a stigma anymore. Academic institutions have also recently developed principles and structures to promote work–life integration for their academic faculty (5). In my institution, a Workload/Worklife task force established jointly by the University and the Association of Academic Staff in 2009 identified that two key actions were to “promote programs, processes, and resources to support staff in meeting challenges of work–life balance” and to “ensure the clarity of job expectations and the alignment of reward systems.” Our University has subsequently established Health Promotion and WorkLife Services to develop workshops for the employees and templates to help individual departments to improve conditions. In addition, even in institutions where these principles and resources exist, I am not aware of convincing evidence that they have been successful.

Excessive work demands because of intensive or long working hours seem also to be a consistent predictor of poor work–life balance (6). In addition, conflicting demands and low level of control over workload can lead to increased stress-related morbidity and loss of balance in ones life. I believe that most, if not all, academic physicians have stressful jobs and experience long working hours, and I certainly have had this experience for the most part of my career. Stress and requirement for excessive time commitment is not explained by on call hours that I am lucky to have very few in my current job, but by hours required in order to fulfill all the roles of my job. However, I also believe we as academic physicians belong to a relatively privileged group. As an academic physician, I have quite a lot of control over the organization of my workday and the activities required for my job. Even the dreaded administrative load was not something that was imposed on my, but something I decided to do thinking that I could be of help for my colleagues and the University in general. As an academic physician, I do something that I like and I can usually do it in my own pace and using my own approaches, although reward depends on how effective I am on my job. This control over our work–life can be lost when demands for certain parts of our job accumulate, but I still believe that we are in a much better position than most other professions.

When I review short periods of my life, a week, a month, or at times even a whole semester, I often notice that there has been no work–life balance, usually for problems such as those described above. For example, when I am preparing to submit a new grant or I am approaching a deadline for a big review, I agreed to write or for a report required by my job (and by the way, I am always a last-minute person, I had to ask for quite a long extension to be able to submit this narrative), I may have to work 10 or more hours a day, 6 or 7 days a week. However, I always know that this is temporary. After I submit the grant, the manuscript, or the report, I am able to compensate for the time lost from my “life.” I will take it easier for a few days, or at times, I may take a few days off and get immersed in other things I like to do, such as traveling or just walking around with my camera(s) in my hands taking pictures or just thinking.

I have to admit that I may not have good answers of how to achieve work–life balance. I know what work–life balance is for me, but as for teaching, my approach to others or helping my students getting a handle on it, I am not sure I am a great help. I always question whether it is possible to transmit your experience to your trainees or whether you can help them with your anecdotes. I have many of these anecdotes and give them out freely to my trainees, even when they do not appear to appreciate them. I also try to show by example that there are ways to overcome the problems that work can impose on you, and that meaningful interactions with colleagues and friends at work and outside work are important for a sense of balance. One regret that I have is that I have not been able to fully reproduce the conditions that I experienced in the first lab I ever worked in Greece, which I believe would have been the best help I could have offered to my trainees and junior colleagues. I hope that the same way I found something useful in the “teachings,” anecdotes, and examples of my mentors, they will also find something among all their interactions with me that can help them later in their career. However, in the end, I tell my students that they would have to develop their own personal approach to work–life balance.

## Author Contributions

The author confirms being the sole contributor of this work and approved it for publication.

## Conflict of Interest Statement

The author declares that the research was conducted in the absence of any commercial or financial relationships that could be construed as a potential conflict of interest.
